# Biological, genomic, and biogeographic characterization of two lytic phages infecting *Vibrio natriegens*

**DOI:** 10.1186/s12985-026-03259-2

**Published:** 2026-07-29

**Authors:** Michiko Takahashi

**Affiliations:** Microbial Systems Research Team, Biomanufacturing Process Research Center, National Institute of Advanced Industrial Science and Technology, Higashi 1-1-1, Tsukuba, 305-8566 Ibaraki Japan

**Keywords:** *Vibrio natriegens*, Bacteriophage, Phylogenetic analysis, Biological characterization

## Abstract

**Background:**

*Vibrio natriegens* is a marine bacterium known for its exceptionally rapid growth and has recently attracted considerable attention as an alternative host microorganism in the field of biotechnology. Despite this growing interest, phages infecting this species remain poorly characterized. In this study, two lytic phages that infect *V. natriegens*, vB_Vna_AM1 and vB_Vna_AM2, were isolated and subjected to biological characterization and phylogenetic analysis.

**Methods:**

Phages vB_Vna_AM1 and vB_Vna_AM2 were isolated from the Sumiyō River mangrove area in Amamigunto National Park, Kagoshima, Japan. Their morphology was determined using transmission electron microscopy. One-step growth curve experiments, host-range evaluation, and viability tests under different temperature, salinity, and pH conditions were performed. Whole-genome sequencing, phylogenetic analysis, and homology searches against global ocean virome datasets were conducted.

**Results:**

vB_Vna_AM1 and vB_Vna_AM2 exhibited prolate capsids and contractile tails, with latent periods of 40–60 min, and both infected *V. natriegens* specifically among the tested strains. Their genome sizes and GC contents were 244,321 bp and 41.51% for vB_Vna_AM1, and 327,464 bp and 40.67% for vB_Vna_AM2, respectively. Both phages were assigned to a subcluster of other vibriophages, showing similarities of 92.1–92.2% for vB_Vna_AM1 and 86.8–86.9% for vB_Vna_AM2. Homology searches using global ocean virome datasets revealed that homologous genes were distributed globally at the protein level; however, they were detected as partial genes within otherwise diverse viral genomes.

**Conclusions:**

This study provides the initial description and phylogenetic analysis of newly isolated phages infecting *V. natriegens*. These findings expand the currently limited repertoire of culturable phages of *V. natriegens* and support the concept that gene modules may be shared among diverse phages within a gene-sharing network, contributing to the understanding of marine viral diversity.

**Supplementary Information:**

The online version contains supplementary material available at https://doi.org/10.1186/s12985-026-03259-2.

## Background

The genus *Vibrio* comprises a diverse group of bacteria that inhabit marine, brackish, and freshwater environments. These bacteria are abundant in aquatic ecosystems, particularly in coastal regions [1]. Among them, several species, including *Vibrio cholerae* and *Vibrio parahaemolyticus*, are well-known human pathogens. In addition, several *Vibrio* species, such as *Vibrio anguillarum*, *Vibrio harveyi*, and *Vibrio parahaemolyticus*, are major pathogens in fish and shrimp aquaculture, causing significant economic losses worldwide [2, 3].

Among these species, *Vibrio natriegens* has recently gained attention from the biotechnology and synthetic biology communities because of its exceptionally rapid growth rate, with a doubling time of less than 10 min [4], which is the fastest reported for any known organism [5]. This characteristic makes it a promising host for metabolic engineering and the large-scale production of various biomolecules [6, 7].

Bacteriophages are viruses that specifically infect bacteria and are the most abundant biological entities on Earth [8]. Numerous phages infecting pathogenic *Vibrio* species have been isolated with the aim of controlling human and aquaculture-associated pathogens, particularly in the context of phage therapy [9]. Phage therapy has recently attracted attention as an alternative to conventional antibiotics because of the emergence of antibiotic-resistant bacteria [10]; however, little is known about phages associated with non-pathogenic but industrially relevant species, such as *V. natriegens*. Only a limited number of *V. natriegens* phages have been described [11]; therefore, the understanding of their diversity, evolutionary relationships, and potential utility as tools for host manipulation remains incomplete.

Here, two lytic bacteriophages infecting *V. natriegens*, designated vB_Vna_AM1 and vB_Vna_AM2, were isolated, and their infection dynamics and genomic features were characterized. The isolates exhibited T4-like morphology and were phylogenetically placed as a sister lineage to known vibriophages. Comparisons with marine virome datasets revealed that genes homologous to vB_Vna_AM1 and vB_Vna_AM2 are widely distributed across marine viromes, whereas genome-level similarity to the isolated phages is limited, suggesting that some gene modules may be shared among diverse phages. These findings expand the previously limited repertoire of characterized phages that infect *V. natriegens* and link culture-based phage characterization with global marine virome datasets.

## Methods

### Bacterial strains and culture conditions

*V. natriegens* NBRC15636, as the isolation host, and *V. alginolyticus* NBRC15630, *V. campbellii* NBRC15631, and *V. harveyi* NBRC15634 were obtained from the National Institute of Technology and Evaluation Biological Resource Center (NBRC), Japan, and routinely cultured in Marine broth (MB) (Sigma-Aldrich, St. Louis, MO, USA) or on Marine agar (MA; MB supplemented with 1.5% agar) at 25 °C. Liquid cultures were shaken at 130 rpm using a reciprocating shaker (BR-23FP; Taitec, Saitama, Japan).

### Phage isolation and purification

Surface water and surface sediment were collected from the Sumiyō River mangrove area in Amamigunto National Park, Kagoshima, Japan, and transported at a constant temperature of 4 °C. Sampling was conducted with permission from the Okinawa Amami Nature Conservation Office, Ministry of the Environment, Japan. The water sample was filtered through a GF/A glass fiber filter (Cytiva, Maidstone, UK) as a prefiltration step, and the filter was incubated in 20 mL MB at 25 °C for 7 days with shaking at 120 rpm. Surface sediment (5 g, wet weight) was suspended in 20 mL MB and incubated under the same conditions. After enrichment, each culture was centrifuged (10,000 ×*g* for 10 min at 4 °C), and the supernatants were filtered through a 0.22-µm membrane filter (Sartorius, Göttingen, Germany). The filtrates were inoculated into exponentially growing *V. natriegens* cultures (OD_600_ = 0.1–0.2) in 96-well plates and incubated overnight at 25 °C. Wells showing lysis were passed through a 0.22-µm filter column (Corning, NY, USA) by centrifugation at 8,000 ×*g* for 10 min at 4 °C. The filtrates were then serially diluted 10-fold, and 50 µL of each dilution was inoculated into a preculture of *V. natriegens* (OD_600_ = 0.1–0.2, 150 µL) in 96-well plates. After incubation at 25 °C with shaking overnight, the extinction-dilution procedure was repeated using the lysates from the most diluted well. Through this procedure, two phage isolates were obtained: vB_Vna_AM1 from the surface water and vB_Vna_AM2 from the sediment.

### Large-scale cultivation of isolated phages

For morphological and genomic analyses, large-scale cultivation was performed according to a previously described method [12] with minor modifications. Briefly, phage lysates were each co-cultured with a preculture of *V. natriegens* in 1 L of MB at a multiplicity of infection (MOI) of 1. After incubation at 25 °C for 4 h with shaking at 130 rpm, the cell lysate was centrifuged at 10,000 *×g* for 5 min at 4 °C to remove cell debris, and the supernatant was subjected to polyethylene glycol (PEG) precipitation. PEG 8000 and NaCl were added to the resulting supernatant to final concentrations of 10% (*w/v*) and 1 M, respectively, and the mixture was incubated at 4 °C overnight. After centrifugation at 10,000 ×*g* for 50 min at 4 °C, the pellet was dissolved in 1 mL of Tris-MgCl_2_ (TM) buffer (10 mM Tris-HCl [pH 7.5] and 5 mM MgCl_2_).

### TEM observation

The phage suspension obtained after PEG precipitation was purified by sucrose density gradient ultracentrifugation. Samples were loaded onto a 10–40% (*w/v*) sucrose step gradient and centrifuged at 100,000 *×g* for 2 h at 4 °C using a P32ST2 rotor in a Himac CP100NX ultracentrifuge (Eppendorf Himac Technologies, Ibaraki, Japan). After centrifugation, the visible phage bands were collected, diluted in TM buffer, and pelleted by ultracentrifugation at 28,000 rpm for 4 h at 4 °C. The resulting pellets were resuspended in TM buffer.

For transmission electron microscopy analysis, a drop of the purified phage suspension was adsorbed onto a carbon-coated Cu grid, negatively stained with 2% phosphotungstic acid for 30 s, and observed using a JEM-1400Plus transmission electron microscope (TEM) (JEOL, Tokyo, Japan) at 100 kV. TEM observation was performed by Tokai Electron Microscopy (Aichi, Japan).

### Phenotypic analysis

The one-step phage growth experiments were performed as previously described [13] with minor modifications. vB_Vna_AM1 or vB_Vna_AM2 was added to an exponentially growing culture of *V. natriegens* at an OD_600_ of 0.1–0.2 (approximately 1 × 10^8^ CFU/mL) at an MOI of 0.1. The mixture was incubated at 25 °C for 5 min with shaking at 130 rpm to allow phage adsorption. The culture was then immediately centrifuged at 1,000 ×*g* for 5 min, and the supernatant was discarded. The cell pellet was resuspended in 1 mL of MB to remove unadsorbed phages, after which incubation was resumed at 25 °C with shaking at 130 rpm. Samples were collected at each time point (0, 10, 20, 30, 40, 50, 60, and 80 min post-infection), and phage titers were determined by plaque assay as described below. The latent period was defined as the time interval between adsorption and the onset of the first burst.

To investigate thermal stability, phage suspensions (approximately 1 × 10^8^ plaque-forming units (PFU)/mL) were incubated at 25, 37, 45, 55, and 65 °C for 60 min. To assess salinity stability, sterilized artificial seawater (Daigo’s Artificial Seawater SP, Shiotani MS, Tokyo, Japan) was diluted with distilled water to prepare solutions corresponding to 0×, 0.25×, 0.5×, and 1× artificial seawater. For pH stability assays, artificial seawater (pH 8.0) was adjusted to pH 3.0, 6.0, and 10.0 using HCl or NaOH. Aliquots of each solution (500 µL) were mixed with 50 µL of phage suspension and incubated at room temperature for 60 min. After each treatment, the samples were immediately serially diluted 10-fold in MB, and phage titers were determined using a plaque assay as described below.

The host range of the phages was tested using a lytic assay against the *Vibrio* strains described above. A 100-µL aliquot of host preculture (approximately 1 × 10^9^ CFU/mL) was mixed with top agar (8 mL of MB supplemented with 0.5% agar) on MA plates. After solidification, 10 µL of phage suspension was spotted onto the top agar. After incubation at 25 °C overnight, the presence or absence of lytic zones was recorded.

### Phage titration

Phage titers were determined by plaque assay according to previously described methods [12, 14] with minor modifications. Briefly, 100 µL of each serially 10-fold diluted phage suspension and 100 µL of *V. natriegens* preculture (approximately 1 × 10^8^ CFU/mL) were co-cultured on double-layered plates. After incubation at 25 °C overnight, the number of plaques was counted. Each experiment was performed with three independent biological replicates. Significant differences in phage titers were analyzed using Tukey’s multiple comparisons test in GraphPad Prism version 10.4.2 for Windows (GraphPad Software, Boston, MA, USA).

### Genome sequencing, assembly, and phylogenetic analysis

The genomic DNA of the isolated phages was extracted using the Maxwell RSC instrument (Promega, Madison, WI, USA) with the Maxwell RSC Genomic DNA Kit (Promega) according to the manufacturer’s instructions. Genomic DNA was quantified using a Quantus Fluorometer with the QuantiFluor dsDNA System (Promega). A sequencing library was prepared using the Ligation Sequencing Kit (SQK-LSK114, Oxford Nanopore Technologies, Oxford, UK) and Flongle Sequencing Expansion (EXP-FSE002, Oxford Nanopore Technologies) according to the manufacturer’s instructions. Each library was sequenced using a Flongle flow cell (FLO-FLG114, Oxford Nanopore Technologies) on a MinION Mk1B with MinKNOW software v24.06.5 (Oxford Nanopore Technologies). Real-time base calling was performed in the fast accuracy modes of Dorado v7.4.12 in the MinKNOW software with the ‘--min-qscore 8’ option. The base-called reads were filtered using a 3-kb length cutoff in the SeqKit [15]. The filtered long reads were *de novo* assembled using Flye v2.9.3 [16] with ‘--isolate’ option. Gene annotation and proteomic tree construction were performed using VipTree version 4.0 [17]. Genome map visualization was performed using gbdraw [18].

### Global distribution of vB_Vna_AM1- and vB_Vna_AM2-like phages

To investigate the global distribution of vB_Vna_AM1- and vB_Vna_AM2-like phages, protein-level similarity searches were performed against large-scale marine virome datasets. Viral contigs from the Global Ocean Viromes 2.0 (GOV2.0) dataset [19] were downloaded from public repositories, and open reading frames (ORFs) were predicted using Prodigal v2.6.3 in metagenomic mode. The predicted ORFs were compiled into a DIAMOND database using DIAMOND v2.1.11.165 [20]. Similarity searches were performed using DIAMOND BLASTP with the following thresholds: E-value ≤ 1e − 5, amino acid identity ≥ 40%, and query coverage ≥ 30%, identifying GOV2.0 viral ORFs homologous to vB_Vna_AM1 or vB_Vna_AM2. To assess the geographic distribution, sampling stations were classified based on the presence or absence of at least one homologous hit, and station locations obtained from the GOV2.0 metadata were plotted on a global map.

Viral contigs containing ORFs homologous to vB_Vna_AM1 or vB_Vna_AM2, identified by DIAMOND, were extracted and used as subjects for nucleotide-level similarity searches. The genomes of vB_Vna_AM1 and vB_Vna_AM2 were used as queries in tBLASTx searches against these contigs. Genomic coverage was calculated as the proportion of the vB_Vna_AM1 or vB_Vna_AM2 genome covered by tBLASTx alignments for each viral contig. Overlapping alignment regions were merged prior to coverage calculation to avoid double counting.

## Results

### Phage isolation and morphology

**Fig. 1:**
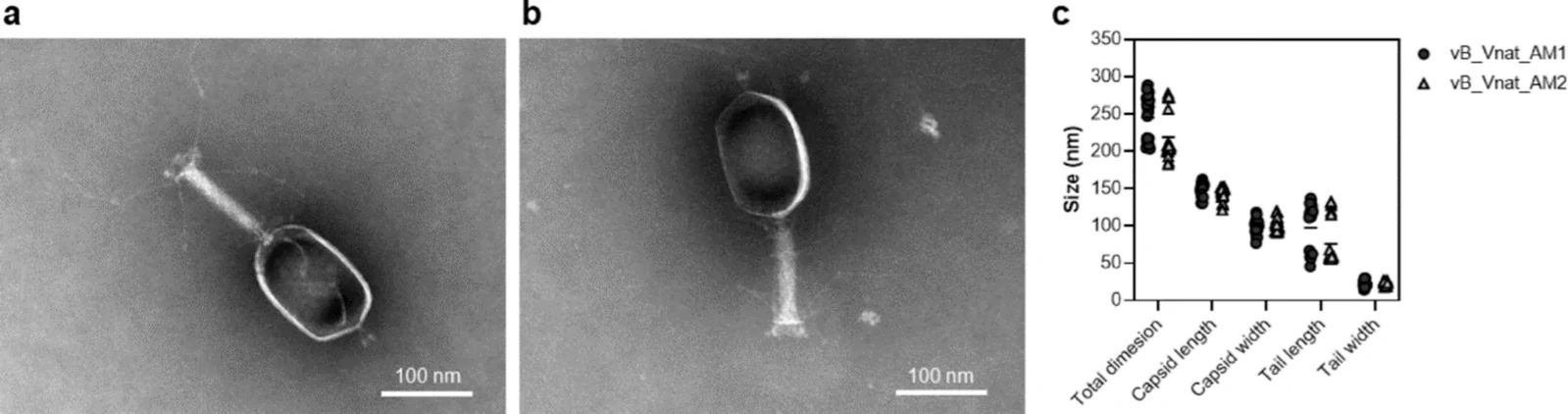
Morphological characterization of isolated phages. Transmission electron microscopy images of vB_Vna_AM1 (**a**) and vB_Vna_AM2 (**b**). Measurements of particle size for each phage (**c**). Size data are shown as mean ± standard deviation (*n* = 20)

Two lytic bacteriophages infecting *V. natriegens*, designated vB_Vna_AM1 and vB_Vna_AM2, were isolated from surface water and surface sediment samples collected in the Sumiyō River mangrove area using an extinction-dilution approach. TEM analysis revealed that both phages possessed prolate capsids and long contractile tails with tail fibers (Fig. 1a and b). The capsid dimensions of vB_Vna_AM1 were 148.33 ± 9.40 nm in length and 100.24 ± 10.01 nm in width, whereas those of vB_Vna_AM2 were 143.87 ± 8.66 nm in length and 103.25 ± 8.87 nm in width. The tail lengths were 97.49 ± 33.20 nm for vB_Vna_AM1 and 75.58 ± 29.77 nm for vB_Vna_AM2 (Fig. 1c). Based on these morphological features, both isolated phages were classified as members of the class *Caudoviricetes*.

### Infection dynamics

**Fig. 2:**
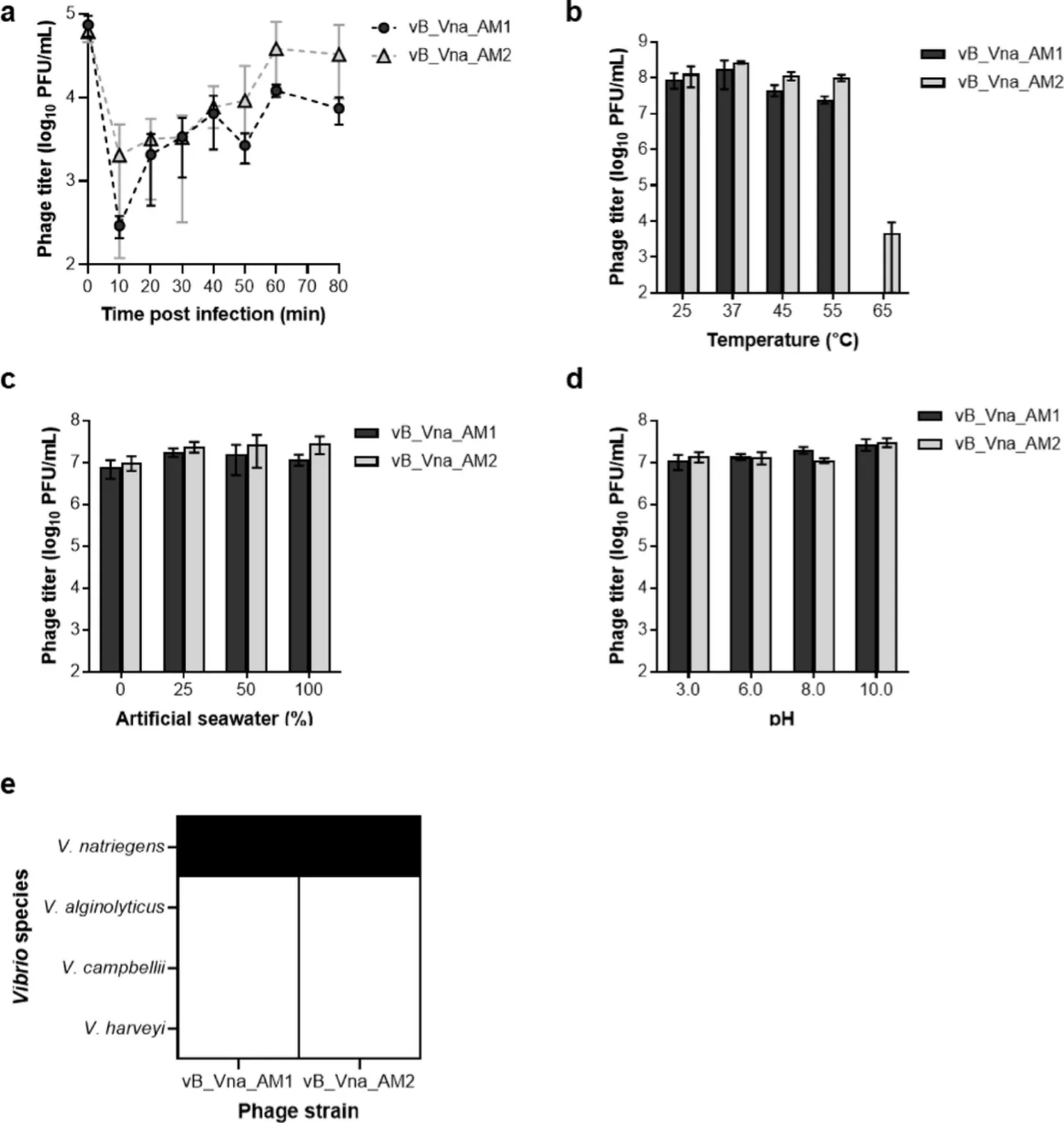
Biological characterization of isolated phages. (**a**) One-step growth curves. (**b**) Thermal stability at 25 °C, 37 °C, 45 °C, 55 °C, and 65 °C for 60 min. (**c**) Salinity stability at 0% (distilled water), 25%, 50%, and 100% artificial seawater (ASW). (**d**) pH stability in artificial seawater adjusted to pH 3.0, 6.0, 8.0, and 10.0. Values represent the mean ± standard deviation (*n* = 3). (**e**) Host range analysis of the isolated phages against *Vibrio* strains

The one-step growth curve experiment was performed to determine the latent period of vB_Vna_AM1 and vB_Vna_AM2 using *V. natriegens* as the host (Fig. 2a). At 0 min post-infection (mpi), the phage titer was higher than that measured at 10 mpi for both isolates, suggesting that unadsorbed particles were not completely removed during the adsorption step and/or that a fraction of particles adsorbed to host cells did not contribute to infection. During 10–30 mpi, the phage titers remained at similar levels for both strains. Subsequently, a reproducible increase in titer was observed in all independent trials for vB_Vna_AM1 at 50–60 min, whereas vB_Vna_AM2 showed an earlier increase at 40–50 mpi. Accordingly, the latent period was estimated to be approximately 50–60 min for vB_Vna_AM1 and 40–50 min for vB_Vna_AM2.

The thermal stability of both phages was assessed after incubation at 25, 37, 45, 55, and 65 °C for 60 min (Fig. 2b). For both strains, the initial titer levels of > 7 log_10_ PFU/mL were maintained after incubation at temperatures ranging from 25 to 55 °C, but the titers significantly decreased at 65 °C for both strains. Notably, the infectious titer of vB_Vna_AM1 decreased to below the limit of quantification (2 log_10_ PFU/mL) after incubation at 65 °C for 60 min. Phage viability was evaluated after exposure to different salinity conditions (1×, 0.5×, 0.25×, and 0× artificial seawater) and pH conditions (pH 3.0, 6.0, 8.0, and 10.0) for 60 min. Both phage titers maintained their initial titer levels (> 7 log_10_ PFU/mL) under all tested conditions (Fig. 2c and d). The host range of both phages was examined using several *Vibrio* species. The vB_Vna_AM1 formed clear lytic zones exclusively on *V. natriegens*, whereas no lytic zones were observed on *V. alginolyticus*, *V. campbellii*, or *V. harveyi*. Similarly, vB_Vna_AM2 showed no lytic activity against these species (Fig. 2e).

### Genome analysis of *V. natriegens* phages

**Fig. 3:**
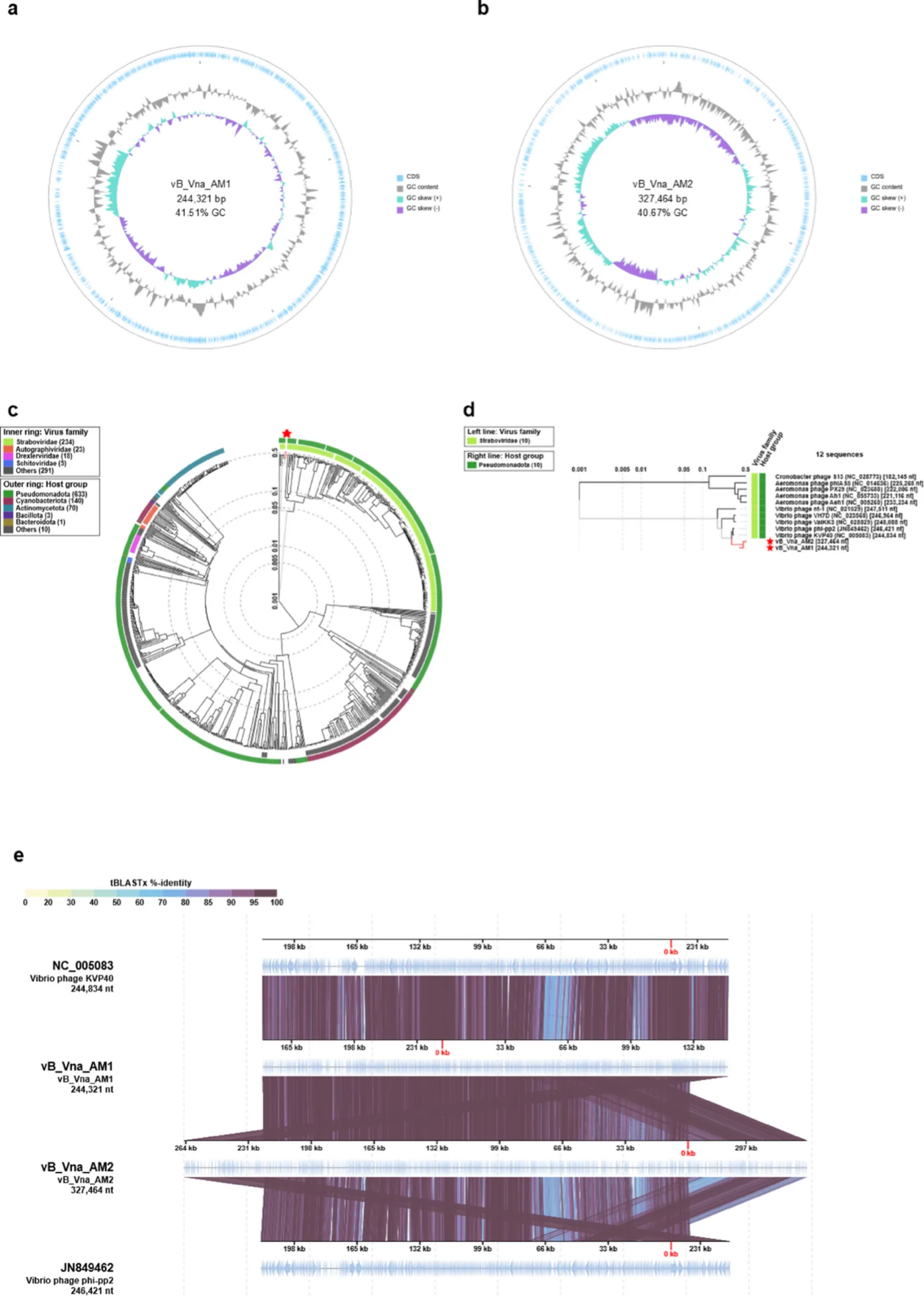
Genome maps, proteomic trees, and genome alignments of isolated phages. (**a**) Genome organization of vB_Vna_AM1. (**b**) Genome organization of vB_Vna_AM2. (**c**) Whole-proteomic tree including 5,632 prokaryotic dsDNA viruses generated by VipTree version 4.0. The dendrogram represents the proteome-wide similarity relationships calculated from the genomic similarity score (Sg) among the two phages isolated in this study and reference viral genomes. Branch lengths are shown on a logarithmic scale from the root of the entire tree. The inner and outer rings of the dendrogram represent viral family classifications and taxonomic groups of known hosts, respectively. (**d**) Expanded view of the proteomic tree featuring the top 10 reference viruses with the highest Sg score, as determined by normalized tBLASTx similarity using VipTree. (**e**) Genome-wide comparison between the isolated phages and their relatives. All alignments are represented by colored lines showing the tBLASTx identities between two viral genomes

The complete genomes of vB_Vna_AM1 and vB_Vna_AM2 were sequenced using Oxford Nanopore long-read sequencing and assembled into a single contig (Table S1). The genome sizes of vB_Vna_AM1 and vB_Vna_AM2 were 244,321 bp and 327,464 bp, respectively, and their GC contents were 41.51% and 40.67%, respectively (Fig. 3a and b). The genome of vB_Vna_AM1 encoded 658 predicted protein-coding sequences (CDSs), of which 625 showed similarity to proteins in the NCBI non-redundant (NR) database, whereas 33 CDSs had no detectable homologs. Similarly, vB_Vna_AM2 encoded 713 predicted CDSs, including 641 NR-hit CDSs and 72 CDSs without detectable homologs.

Proteome-based phylogenetic analysis using the VipTree server placed both phages within a clade comprising members of the family *Straboviridae* (Fig. 3c). An expanded view of this region revealed that vB_Vna_AM1 and vB_Vna_AM2 formed a subcluster, representing a sister lineage to the cluster containing Vibriophage KVP40 and Vibriophage phi-pp2 (Fig. 3d) [11, 21]. vB_Vna_AM1 showed tBLASTx identities of 92.1% and 92.2% to KVP40 and phi-pp2, respectively, whereas vB_Vna_AM2 exhibited lower identities of 86.9% to KVP40 and 86.8% to phi-pp2. Genome-wide comparisons between the isolated phages and their closest relatives (Vibriophage KVP40 and phipp2) showed variable regions containing tail fiber, DNA primase, and hypothetical protein genes (Fig. 3e).

### Global distribution of vB_Vna_AM1- and vB_Vna_AM2-like phages

**Fig. 4:**
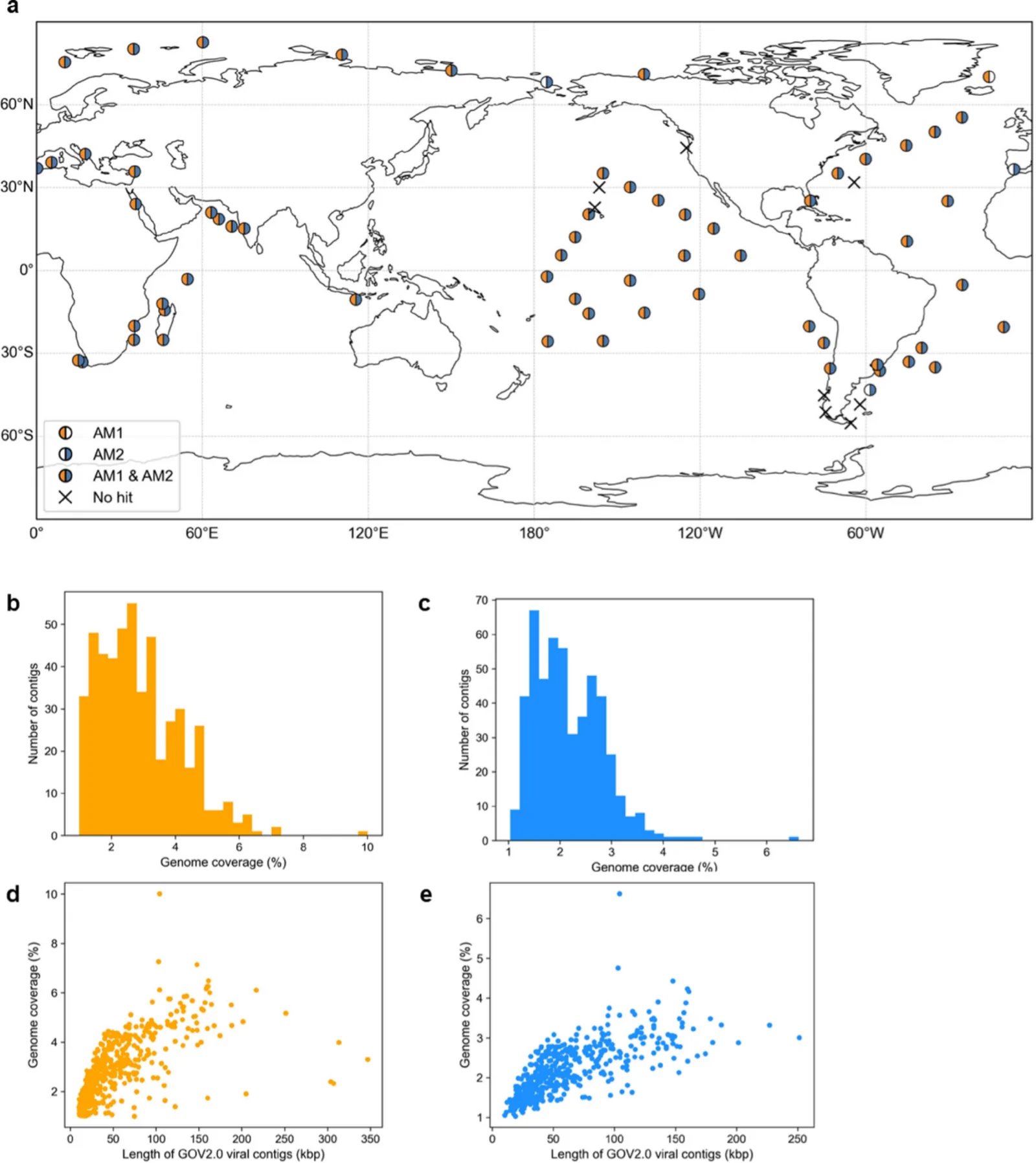
Global distribution of vB_Vna_AM1- and vB_Vna_AM2-homologous genes and genome-level similarity to ocean viral contigs. (**a**) Global distribution of genes homologous to vB_Vna_AM1 and vB_Vna_AM2 in marine viromes. Homologous proteins were identified through DIAMOND BLASTP searches of vB_Vna_AM1 and vB_Vna_AM2 ORFs against the Global Ocean Viromes 2.0 (GOV2.0) viral ORF dataset. Each circle represents a sampling station included in the GOV2.0 dataset. Orange semicircles indicate stations where vB_Vna_AM1-homologous genes were detected, blue semicircles indicate stations where vB_Vna_AM2 homologous genes were detected, and circles split into orange and blue indicate stations where homologous genes to both phages were detected. Crosses denote stations where no homologous genes were identified. (**b**,** c**) Genome coverage of vB_Vna_AM1 (**b**) and vB_Vna_AM2 (**c**) by GOV2 viral contigs containing homologous genes. Genome coverage was calculated from tBLASTx alignments between the vB_Vna_AM1 or vB_Vna_AM2 genome and the corresponding GOV2 viral contigs. Coverage represents the fraction of the AM1 or AM2 genome covered by merged alignment regions for each contig. (**d**,** e**) Relationship between the length of GOV2 viral contigs and the genome coverage of vB_Vna_AM1 (**d**) or vB_Vna_AM2 (**e**)

To explore the global distribution of genes homologous to vB_Vna_AM1 and vB_Vna_AM2, homology searches were performed against the Global Ocean Viromes 2.0 (GOV2.0) dataset using DIAMOND BLASTP. Homologous genes were detected at 64 of 72 sampling locations (89%) in the dataset (Fig. 4a). Among these locations, 60 contained homologs to both vB_Vna_AM1 and vB_Vna_AM2, whereas one location contained only vB_Vna_AM1 homologs and three locations contained only vB_Vna_AM2 homologs. The datasets from the remaining eight locations did not contain homologous genes. Functional classification of vB_Vna_AM1- and vB_Vna_AM2-derived ORFs homologous to GOV2.0 viral contigs revealed that the most abundant functional category was DNA replication, recombination, and repair (27,878 hits), followed by nucleotide metabolism and auxiliary metabolism (12,189 hits), and head and packaging (7,345 hits) (Tables S2 and S3).

To evaluate genome-level similarity between these marine viral sequences and the isolated phages, viral contigs containing homologous genes were compared with the vB_Vna_AM1 and vB_Vna_AM2 genomes using tBLASTx. Across the viral contigs analyzed for each phage, the median genome coverage was 2.69% for vB_Vna_AM1 and 2.05% for vB_Vna_AM2, with mean values of 2.91% and 2.16%, respectively. The maximum coverage was 10.0% for vB_Vna_AM1 and 6.6% for vB_Vna_AM2, indicating that most contigs shared only a small fraction of the phage genomes (Fig. 4b and c). Although genome coverage increased with the length of GOV2.0 viral contigs (Pearson’s *r* = 0.65 for vB_Vna_AM1 and *r* = 0.73 for vB_Vna_AM2), the maximum coverage remained 10.0% for vB_Vna_AM1 and 6.6% for vB_Vna_AM2 (Fig. 4d and e).

## Discussion

Despite its ecological and biotechnological relevance, the diversity, infection dynamics, and environmental distribution of phages infecting *V. natriegens* remain largely unexplored. In this study, two novel lytic bacteriophages infecting *V. natriegens*, vB_Vna_AM1 and vB_Vna_AM2, were isolated and characterized, thereby adding to the number of described phages and linking isolate-level characterization to global ocean viromes.

The latent periods of the isolates were approximately 50–60 min for vB_Vna_AM1 and 40–50 min for vB_Vna_AM2 (Fig. 2a). The phage titers at 0 mpi were higher than those at 10–30 mpi. Possible explanations for this include incomplete removal of unadsorbed particles during the adsorption step; alternatively, some adsorbed particles may not have resulted in productive infection but were nevertheless detected in the initial titer measurements. As this pattern complicates the accurate determination of the number of infective particles present immediately after adsorption, reliable estimation of the burst size was not possible in the present study. Therefore, the one-step growth experiments were used primarily to estimate the latent periods. Both phages retained infectivity at temperatures up to 55 °C and remained stable across a broad range of salinity and pH conditions. Such tolerance to multiple environmental stressors may facilitate persistence in fluctuating coastal environments (Fig. 2b, c, and d).

Both phages exhibited a T4-like morphology characterized by a prolate head and a contractile tail (Fig. 1a and b), a morphotype characteristic of phages in the family *Straboviridae*. This classification was further supported by the phylogenetic analysis results (Fig. 3c). Comparative genomic analyses indicated that the closest relatives of the present isolates were phages KVP40 and phi-pp2: KVP40 can infect eight *Vibrio* species and one *Photobacterium* species, whereas phi-pp2 can infect *V. parahaemolyticus* and *V. alginolyticus* [11, 21]. By contrast, the host-range assays revealed that both vB_Vna_AM1 and vB_Vna_AM2 infected only *V. natriegens* among the tested strains (Fig. 2e). Genome alignments among these phages identified variable regions containing genes encoding tail fiber proteins (Fig. 3e). Such variation may reflect differences in receptor recognition during the adsorption step, potentially accounting for the observed differences in host range.

The detection of genes homologous to vB_Vna_AM1 and vB_Vna_AM2 across global marine viromes suggests that homologous genes are widely distributed in marine viral communities (Fig. 4a). However, although genes homologous to vB_Vna_AM1 and vB_Vna_AM2 were widely detected across various marine viromes, genome-level comparisons showed that only limited regions of the marine viral contigs were homologous to the isolated phage genomes (Fig. 4b and e). Viruses share genes among evolutionarily distant lineages through gene-sharing networks [22]. The predominance of homologs associated with DNA replication, recombination, and repair functions (Table S2) is consistent with the notion that these genes are more widely conserved across diverse viral lineages than genes involved in host recognition. Therefore, the genes homologous to vB_Vna_AM1 and vB_Vna_AM2 detected in this study may represent shared functional gene modules distributed among diverse marine viral genomes through such gene-sharing networks.

In this study, the phages infecting *V. natriegens* were successfully isolated from soil and creek water samples after enrichment in liquid culture. Similar enrichment approaches have been used for phage isolation from environmental matrices [23]. In contrast, no phages were recovered from the viral fractions (0.2 μm filtrates) of water or sediment suspensions (data not shown), suggesting that the abundance of infectious particles in the original samples was likely below the detection limit under the experimental conditions. Collectively, these results indicate that pre-incubation of environmental samples increases the probability of phage isolation. However, such procedures introduce selection bias, as phages unable to propagate under laboratory conditions are likely to be overlooked.

## Conclusions

In summary, the findings of the present study expand the currently limited repertoire of characterized phages infecting *V. natriegens* and provide novel insights into their ecological distribution and genomic relationships to environmental viral sequences.

## Supplementary Information

Below is the link to the electronic supplementary material.

Supplementary Material 1

Supplementary Material 2

Supplementary Material 3

## Data Availability

The assembled genomes have been deposited in the DDBJ/ENA/GenBank databases under accession numbers listed in Table S1. All data are associated with BioProject PRJDB40651.

## Abbreviations

MB: Marine broth
MOI: Multiplicity of infection
PEG: Polyethylene glycol
TEM: Transmission electron microscopy
ORF: Open reading frames
CDS: Coding sequence
NR: Non-redundant
